# Understanding the Impacts of Novel Coronavirus Outbreaks on People Who Use Drugs: A Systematic Review to Inform Practice and Drug Policy Responses to COVID-19

**DOI:** 10.3390/ijerph18168470

**Published:** 2021-08-11

**Authors:** Alison Munro, Hazel Booth, Nicola M. Gray, Joan Love, Andrea R. M. Mohan, Jason Tang, Steve MacGillivray

**Affiliations:** 1 Drug Harms Prevention Research Group, School of Health Sciences, University of Dundee, Dundee DD1 6HN, UK; n.y.gray@dundee.ac.uk (N.M.G.); JLove001@dundee.ac.uk (J.L.); AMohan001@dundee.ac.uk (A.R.M.M.); jason.tang@stir.ac.uk (J.T.); s.a.macgillivray@dundee.ac.uk (S.M.); 2 Faculty of Social Sciences, University of Stirling, Stirling FK9 4LA, UK; h.l.booth@stir.ac.uk

**Keywords:** COVID-19, coronavirus, SARS, MERS, people who use drugs, problem drug users, service responses, systematic review

## Abstract

People who use drugs (PWUD) experience many social and health harms and are considered at greater risk of acquiring COVID-19. Little research has examined the impact of coronaviruses either on PWUD, or on services targeted to PWUD. We report the findings of a systematic review of empirical evidence from studies which have examined the impact of coronaviruses (Severe Acute Respiratory Syndrome (SARS-CoV-1) and Middle Eastern Respiratory Syndrome (MERS-CoV) and COVID-19) on PWUD or on service responses to them. Five databases were searched (MEDLINE, PsycINFO, CINAHL, ASSIA and EMBASE) as well as COVID-19 specific databases. Inclusion criteria were studies reporting any impact of SARS, MERS or COVID-19 or any service responses to those, published between January 2000 and October 2020. Weight of Evidence judgements and quality assessment were undertaken. In total, 27 primary studies were included and grouped by seven main themes: treatment/recovery services; emergency medical settings; low-threshold services; prison setting, PWUD/substance use disorder (SUD) diagnosis; people with SUD and HIV; ‘Sexual minority’ men. Overall, research in the area was scant, and of average/poor quality. More robust research is required to inform on-going and future responses to coronavirus epidemics for PWUD.

## 1. Introduction

Globally, there are an estimated 35.6 million people experiencing ‘drug use disorders’ [1] (defined as ‘intoxication by, dependence on, or regular, excessive consumption of psychoactive substances’) [2]. Although the most common used drug globally is cannabis and indeed drug markets are changing in terms of increased use of amphetamines and of synthetic substances, those that are associated with most health-related harm are opioids [1]. Approximately 58 million people use opioids and opioid users accounted for 66% of over 167,000 drug-related deaths (DRDs) in 2018 [1]. As well as DRDs, people who use drugs (PWUD), experience multiple social and health harms, including blood borne viruses (BBV) infections and are, in general, at elevated risk of premature mortality compared to other groups [3]. DRDs have themselves been described as having reached ‘epidemic’ proportions in some countries including the United States of America (USA) and Canada [4], and in Scotland (UK) as a public health emergency [5]. Within the UK, Scotland has the highest rate of DRDs of any European country at 0.18 per 1000 (or 18 per 100,000) of the population [6]. In the USA and Canada, where prescription opioid use is a major contributor to DRDs, the respective rates were 21.6 per 100,000 in 2020 [7] and 10.3 per 100,000 (opioid related deaths only) in 2019 [4]. 

Factors that are known to contribute to DRDs vary between countries but include increases in use of prescription opioids in North America [8] and poly-substance use, concurrent benzodiazepine use and an ageing population of people with complex needs in Scotland [6]. Additionally, however, structural factors such as poverty, stigma and economic and social deprivation impact on the lives of PWUD and on the harms they experience in regard to their health and wellbeing, including DRDs [9,10,11]. People who experience problems with drug use also often have a higher prevalence of physical and mental health issues than the general population [12,13]. Physical problems include cardiovascular disease [14], respiratory illnesses [15], pulmonary disease, [16] asthma and diabetes, as well as the impacts of BBVs [3]. Unfortunately, many of these illnesses are some of the key ones which, if pre-existing in individuals, can make them more susceptible to having poorer outcomes if they acquire COVID-19 [17,18]. 

COVID-19, a type of coronavirus, is a disease caused by severe acute respiratory syndrome coronavirus 2 (SARS-CoV-2) [19]. It was first recorded in December 2019 and has since become a global pandemic (declared by the World Health Organization on 11 March 2020) which already (at the time of writing) has infected over 120 million people and led to more than 2.6 million deaths [20]). The countries with the highest rate of deaths per 100,000 population from COVID-19 include the Czech Republic, the UK, Hungary, Italy and the USA [20]. 

Prior coronavirus outbreaks that include both the Severe Acute Respiratory Syndrome (SARS-CoV-1) in 2002 and Middle Eastern Respiratory Syndrome (MERS-CoV) in 2012 led to 1602 deaths in total [21]; the largest proportion of whom were males and between 41 and 60 years old [22]. Data that were published in the early stages of the COVID-19 pandemic showed that specific groups of people appeared to be at elevated risk of mortality from COVID-19, e.g., older people, males, those with specific underlying conditions (cardiovascular, pulmonary, diabetes mellitus and asthma) [23] and those from poorer neighbourhoods [24,25]. Ongoing research both on the prevalence and impact of COVID-19 have continued to confirm these early data [26] as well highlighting the higher risk of contracting the virus amongst people from black and minority ethnic (BAME) communities. Sze et al. [27] provide systematic review level evidence that people who are black and Asian are at higher risk of acquiring COVID-19 and that people from Asian communities are at greater risk of mortality than their White counterparts [27]. Another more recently identified feature of COVID-19 is that many people who have contracted the virus continue to exhibit symptoms of the disease, sometimes very severe, for quite some time afterwards. Researchers, and clinicians, are only beginning to understand the phenomenon of post COVID syndrome (‘long COVID’), defined as ‘signs and symptoms that develop during or following an infection consistent with COVID-19 which continue for more than 12 weeks and are not explained by an alternative diagnosis’ [28]. It is not yet known whether PWUD may be more or less likely than the general population to be at risk of post-COVID syndrome. However, the similarities between the factors that appear to be both predictors of, and have impacts on, people who contract COVID-19 and those that are highly prevalent amongst PWUD are stark, suggesting that PWUD may be at greater risk of mortality (and other harms) from COVID-19 than their non PWUD counterparts [29,30]. This review sought to establish the extent and quality of extant empirical evidence to examine the impacts of SARS-Cov-1, SARS-Cov-2 (COVID-19) and MERS-Cov on PWUD and on service responses to these coronavirus outbreaks of the 21st Century. The specific aim and objectives are outlined below. 

### Aims and Objectives

The aim of this review is to inform current and future responses to coronavirus outbreaks by identifying and critically appraising the empirical evidence relating to the impact of novel coronavirus outbreaks of the 21st Century on problem drug users, with a particular focus on COVID-19, on drug related deaths and other drug-related harms, as well as on service responses to problem drug use (PDU) including any evidence of the effectiveness of such responses. The four review questions were:

1. What evidence exists regarding the impacts of any of the novel coronavirus outbreaks of the 21st Century, including COVID-19, on PWUD, drug-related deaths and other harms and what is the quality of evidence?
2. How have services who provide support for PDU and policy makers responded to any of the 21st Century outbreaks, including COVID-19, and what is the quality of evidence supporting their responses?
3. What are the gaps in evidence and what are the future research questions of importance in responding to any future outbreaks?
4. What are the implications of past responses to coronavirus epidemics/pandemics to inform future service and policy responses?

## 2. Materials and Methods

The protocol for the systematic review was registered with PROSPERO, an international prospective register of systematic reviews and can be accessed online [CRD42020211227]. In addition, the review was conducted in accordance with the Preferred Reporting for Systematic Reviews and Meta-Analyses (PRISMA) guidelines [31]. The completed PRISMA checklist is included as Supplementary File S1.

### 2.1. Search Strategy

The following databases were searched: MEDLINE, PsycINFO, CINAHL, ASSIA, and EMBASE. 

The search strategy adopted the following search architecture:

1. COVID-19 OR CORONAVIRUS OR 2019-ncov
2. SARS-CoV-2
3. SARS-CoV-1
4. MERS
5. SARS
6. 1 OR 2 OR 3 OR 4 OR 5
7. Problem Drug Users OR People who use drugs OR people who inject drugs
8. 6 AND 7
9. Limit 8 to English language; limit 8 to year range 2000–2020

We also conducted a further search which included the use of COVID-19 specific search engines including CoViz, the ResearchGate COVID-19 search community, Lit Covid; COVID-19 EPPI Centre living map of evidence and Evidence Collection from EvidenceAid UK and OpenGrey. Electronic backwards and forwards citation searching of papers identified were also undertaken and we wrote to key authors in the field to ask if they were aware of any recently completed or ongoing studies. The search results were deduplicated and then citations screened according to the criteria detailed below. The full search architecture is given in Supplementary File S2. 

### 2.2. Inclusion and Exclusion Criteria

The included studies focused on problem drug users/people who use drugs or services targeted to PWUD and included studies of any design and reporting any outcome. Full details of the inclusion and exclusion criteria are given in Table 1 below.

### 2.3. Data Extraction and Reporting

All titles and abstracts from the initial list of potential studies were screened independently by two members of the study team to identify studies to be included in full text screening (AM and SM). Where there was disagreement between the researchers, reports were retained for further examination. Selection was based on the above inclusion and exclusion criteria. Two members of the study team independently examined full-text copies of all selected papers for eligibility (AM and SM), and disagreements were resolved by consulting with a third team member (JL). Data were then extracted from the studies selected for inclusion. An Excel spreadsheet was used to record extracted information. 

All included papers were scrutinised, and the following categories of data were extracted by one reviewer (SM): study type; study aims; study methods; study setting; country study took place in; year study took place; study population; main study findings; study conclusions. Data were tabulated according to these categories and then sorted to facilitate descriptive reporting. 

### 2.4. Study Assessment/Quality Assessment

We adopted the Evidence for Policy and Practice Information and Co-ordinating Centre [32] approach to assessing quality and relevance of studies: EPPI-Centre weight of evidence (WoE) judgements were applied to each of the included studies. Three components were assessed to help derive an overall weighting of evidence score: methodological quality, methodological relevance and topic relevance. These components are detailed in Table 2 below.

Assessment of overall weight of evidence (WoE) was made based on the assessments for each of the above criteria and by using the same scoring system.

### 2.5. Additional Quality Assessment

We also conducted a more detailed assessment of the methodological quality of the included studies based upon individual elements of study quality. 

#### 2.5.1. Qualitative Studies

All included qualitative studies were subject to an assessment of individual study quality items, drawing upon Critical Appraisal Skills Program (CASP) [33] and COnsolidated criteria for REporting Qualitative research (COREQ) criteria: triangulation of data, rigor, reflexivity, credibility, relevance, and clear exposition of ethical issues [34]. We also considered the nature of the evidence reported in the qualitative studies and assessed these in terms of the ‘typologies’ of their findings as described by Sandelowski and Barroso (2003) [35]. These authors suggest that findings of qualitative studies can be classified on a continuum of data transformation, from findings that are not qualitative (no finding, topical survey), to ones that are exploratory (thematic survey), descriptive (conceptual/thematic description) or explanatory (interpretive explanation). 

#### 2.5.2. Quantitative Studies

All included questionnaire surveys were subject to an assessment of individual study quality items. Key items of methodological quality of questionnaire surveys assessed were representativeness of the sample (to the population from which they were drawn); validated questionnaire (was a previously validated questionnaire used); response rate; whether or not the study was longitudinal as opposed to cross-sectional; missing data; generalisability of study findings.

#### 2.5.3. Service Evaluation Studies

Those studies that employed a method of service evaluation or an approach to analysing service use via existing data such as electronic health records were assessed against the following four criteria: representativeness of sample studied, prospective rather than retrospective, longitudinal rather than cross-sectional and generalisability of study findings. 

## 3. Results

**Figure 1:**
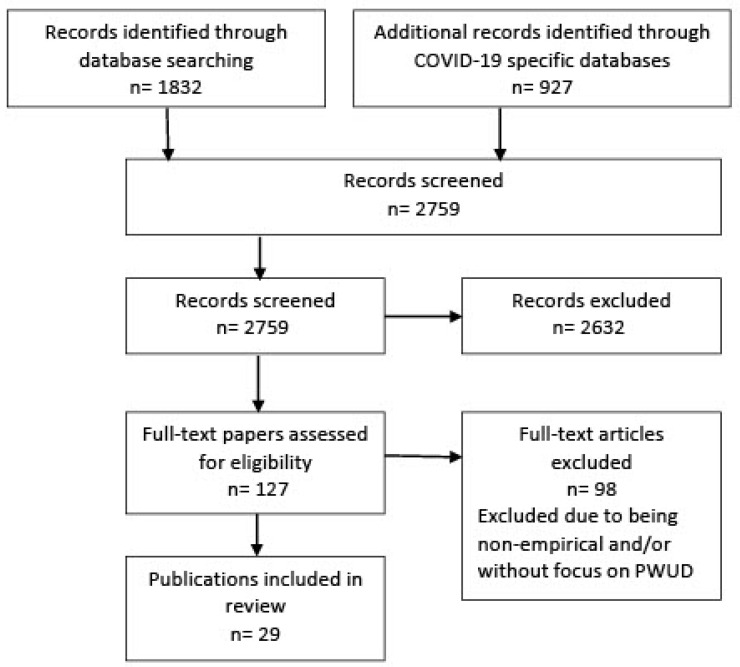
Flow diagram of study selection.

The results of searching multiple sources for literature reporting empirical studies resulted in 2759 publications being screened (see Figure 1 below). Screening by title and abstract resulted in 2632 being excluded due to not fitting inclusion criteria. In total, 127 publications were retained and retrieved in full for further detailed scrutiny. Subsequently, 98 further studies were excluded because they were not reports of an empirical study and/or they did not focus on PWUD. Twenty-seven reports of empirical studies were located. 

### 3.1. Overview of Study Characteristics

Table 3 provides an overview of the 27 primary studies included in the review (ordered by weight of evidence and setting relevance). The majority of studies were conducted in North America (14) and the remaining 13 were conducted in Spain (2) and 1 each in Australia, Finland, India, Italy, Netherlands, Nigeria, Norway, Poland, UK and Ukraine, with the final study involving participants from multiple countries. Twenty-five studies were quantitative and two were qualitative. No studies related to SARS-CoV-1 or MERS. Our analysis of studies indicated that they were best categorised with regard to their focus on treatment setting/services. Seven treatment settings were identified:

- Treatment services/treatment/recovery (8 studies);
- Emergency medical services (5 studies);
- Low threshold services/shelter/homelessness (3 studies);
- People who use drugs/with diagnosis of SUD (7 studies);
- People with SUD and HIV (2 studies);
- Sexual minority CIS men (1 study);
- Prison (1 study).

### 3.2. Quality Assessment 

#### 3.2.1. Weight of Evidence: Methodological Quality, Methodological Relevance and Topic Relevance 

As can be seen from Table 4, below, the methodological quality of included studies was assessed mainly as less than ‘good’. No studies were rated as excellent in terms of methodological quality, only 8 as good and 2 were rated as inadequate. In terms of methodological relevance, ratings were better, with 4 rated as excellent, 9 as good, 13 as satisfactory and 1 as inadequate. In total, 21 of the 27 papers were rated as good or above in terms of topic relevance. However, with regard to overall weight of evidence, the studies as a whole did not rate highly, with only 11 being rated as ‘good’ and the rest as either satisfactory or inadequate. None were rated as excellent. 

#### 3.2.2. The Additional Quality Assessment

The two qualitative studies were assessed as being credible and relevant but were weaker on the other areas of quality assessed, such as rigor and reflexivity. Of the 13 studies employing a survey method, only two reported a response rate of over 60%, and no studies were rated as good for all elements of methodological quality. Of the 12 studies using other quantitative observational methods, only two were longitudinal and none were prospective suggesting limitations in terms of methodological robustness (please see Supplementary File S3 for a summary of quality assessment).

### 3.3. Results Presented by Theme/Setting

As mentioned above, included studies were grouped by treatment/setting for analysis and assessment of quality. The seven key settings are presented and discussed below.

#### 3.3.1. Treatment/Recovery Services (Eight Studies)

The largest group of studies focused on treatment/recovery services (Table 5). Six of the eight studies sought the views of PWUD regarding the impact of the COVID-19 pandemic [36,37,42,53,54,61]. There is good quality evidence that the pandemic has had serious impacts on thoughts, feelings and behaviours such as stress, anger and feelings of isolation. There is varied evidence (of mixed methodological quality) that the pandemic has reduced quality of life amongst PWUD. In addition, one included UK study provides ‘satisfactory’ evidence that the impact of COVID-19 on PWUD was a reduction in Needle and Syringe Program (NSP) clients as well as visits to NSPs during the initial stages of the pandemic [48]. The impact was found to be greatest for those injecting image and performance enhancing drugs. 

#### 3.3.2. Emergency Medical Services (EMS) (Five Studies)

All five of the studies that were set in EMS (Table 6) employed a quantitative method [38,40,44,45,55]. All were pre- and post-studies of PWUD sampled from those who had visited emergency departments or had contact with ambulance services before and after stay-at-home restriction orders, and all were conducted within the USA. Four of these studies ranked as ‘good’ with regard to overall weight of evidence and one was satisfactory; though only three were also rated as ‘good’ in terms of methods. As a group of studies these can be said to constitute the ‘strongest’ evidence in the included papers. 

Three of the five studies suggested that opioid overdoses increased during the COVID-19 stay-at-home order along with increased number of visits to the emergency department for naloxone administration [38,44,55]. Similarly, two studies showed that emergency medicine calls for opioid overdose increased during this time [40,45]. Two studies reported an increase in drug overdose deaths [38,54] but only one of these was rated as ‘good’ in terms of methods used and in weight of evidence. 

#### 3.3.3. Low Threshold Services/Shelter/Homeless (Three Studies)

Two of these three studies employed a quantitative method (Table 7) [39,60]. These were questionnaire surveys of PWUD sampled from homeless populations and those who attended drop-in services. The remaining study was a program evaluation involving structured interviews with homeless people, 33% of whom had a substance use disorder (SUD) [62]. 

Only one of these three studies was judged as ‘good’ overall (WoE), one was ‘satisfactory’ and one was ‘inadequate’ indicating that the evidence around this group/setting is not good overall from included papers. However, the paper that was judged as inadequate in terms of overall WoE was one of the few to include analysis of the use of services throughout the early stages of the pandemic by gender and concluded that service use by women declined more than did service use by men (no analyses conducted using inferential statistics) [60].

The program evaluation suggested that homeless people with a substance misuse disorder may experience greater difficulty accessing a range of health services and treatments. 

One questionnaire survey indicated that those who were enrolled on Opioid Maintenance Treatment (OMT) during the pandemic had greater awareness of the symptoms of COVID-19 as well as access to services that they could attend highlighting the potential importance of OMT programs for COVID-19 prevention [39]. 

#### 3.3.4. People Who Use Drugs/with Diagnosis of Substance Use Disorder in Predominantly Community Settings 

All seven studies used quantitative methods: three were questionnaire based surveys [50,51,57]; one was an assessment of urine samples pre and post COVID-19 [59]; one was a large case–control study of electronic health records [46]; another also analysed electronic health records [56], and the remaining one an analysis of post-mortem toxicology data [41]. Table 8 shows that only two of these studies were judged as ‘good’ in terms of weight of evidence, and the remaining five were judged as satisfactory, meaning caution is needed in interpreting the results.

The study by Wang and colleagues, conducted on data collected across the USA, provides good evidence that people with a diagnosed SUD, and those categorised as African American are at higher risk of contracting COVID-19 as well as adverse outcomes from the virus [46]. 

Three of the remaining six studies, all surveys, suggested that substance use either declined or did not change since the onset of COVID-19 restrictions [50,51,57]. Two of the three surveys report a deterioration in mental health amongst both community and clinical samples [50,51]; one reported reduced access to treatment services [57] and one reported no issues for PWUD in terms of accessing services [51]. However, in contrast, the cross-sectional study (judged ‘good’ in methods used) indicated increased use of the illicit drugs, cocaine, fentanyl, heroin and methamphetamine amongst community samples using data from multiple states [59].

The study by Little and colleagues [56] analysed prescription data of first-time prescriptions for Medications for Opioid Use Disorder (MOUD) and found that these had decreased by over 30% in the spring of 2020 when compared to trends observed in electronic health record data from January 2017 to May 2020.

The final study in this group reported a before and after ‘stay at home orders’ study of post-mortem toxicology cases found to be positive for buprenorphine, amphetamine or cannabis in the first 8 months of the year 2020 [41]. They found that for the period directly after the government restrictions came into force in March 2020, the numbers of positive screening for buprenorphine, amphetamine and cannabis increased in post-mortem data. The increase was most noticeable for amphetamine and was evident in all age groups.

#### 3.3.5. People with HIV and Substance Use Disorder

Both of the studies of people with SUD and HIV were surveys; one was judged as ‘good’ in terms of WoE and one as ‘satisfactory (Table 9)’. Both were judged only as ‘satisfactory’ in terms of methods. One study reported that people with SUD and HIV had increased their illicit drug use and were less confident about staying sober/abstinent and attending recovery services during the pandemic [43]. The other survey of older people with SUD and HIV found that, whilst this group maintained SUD and HIV therapy during the pandemic, social support was critical in avoiding treatment interruptions [58].

#### 3.3.6. ‘Sexual Minority’ Men

Only one study of ‘sexual minority’ cisgender (Cisgender men refers to ‘non transgender’ people [63]) men was included in the review (Table 10). This study employed a matched control design using survey methods and found that while drug use declined amongst this group after the pandemic started, sexual risk-taking behaviour did not [47].

#### 3.3.7. Prison

This single study (Table 11) was a survey of prison staff across various prisons in the USA and found that there were disruptions to medication dispensing processes including medication-assisted treatment (MAT), i.e., Opioid Agonist Treatment (OAT), because of challenges in maintaining social distancing and obtaining personal protective equipment (PPE) at the time. This study was judged as ‘satisfactory’ in terms of both WoE and methods used [49]. 

## 4. Discussion

To the best of our knowledge, this is the first systematic review to report the results of a review of empirical evidence only that was conducted specifically to assess the impact of SARS-1, MERS and COVID-19 on problem drug users. From the 27 empirical papers included, we found that they were best grouped and described under the seven themes listed in the preceding Results Section. This section discusses the key study findings including implications for the ongoing pandemic as well as future coronavirus pandemics.

Although the included studies are limited in number and in quality overall, several discussion points emerge from the study results and cut across the study groupings. These are impact on psychological and physical wellbeing for PWUD including for ‘vulnerable groups’ (e.g., people who were homeless at the time of the pandemic, and BAME groups); restricted access to services and service ‘innovations’ to mitigate some potential restrictions; impacts on drug-related deaths and other harms; drug use patterns themselves. 

### 4.1. Psychological and Physical Wellbeing

Several studies examined the impact of COVID-19 on psychological and physical wellbeing of PWUD and found, perhaps unsurprisingly, that impacts were detrimental and in different ways. While out of the scope of our review, it is clear from research conducted on even general populations that the impact of COVID-19 including the experience of social restrictions has led to increases in both anxiety and depression globally [64]. Additionally, there is increasing evidence that the impact on mental health has been comparatively much worse in other vulnerable groups (such as those in insecure housing, BAME groups, and those with pre-existing mental health conditions, and those with low social support [65,66]. Given that PWUD can be defined themselves as a vulnerable population then it would seem reasonable to suggest that additional supports for both physical and psychological health may need to be put in place, prospectively, if further pandemics such as this occur. 

### 4.2. Vulnerable Groups

Many jurisdictions including countries of the UK and Ireland, Spain and the USA recognised early in the pandemic that people who were homeless would be additionally vulnerable to COVID-19 as well as to drug-related harms if interventions were not in situ place to house them. These studies were few and again of limited quality. However, they highlighted that creating specific measures to support people who were homeless could be effective [62], that being in contact with harm reduction services was an effective way to convey important knowledge and information about COVID-19 [39] and potentially that women who use drugs were more affected by service disruptions than men [60]. In Scotland and the UK, people who were homeless (significant proportions of whom tend to be PWUD) were also provided with temporary accommodation in hotels during the initial wave of the pandemic [67,68] but the outcomes and impacts of these initiatives are currently under-researched (or under-reported) and so the results of these initiatives are not yet known. However, one recent qualitative study in Scotland found that maintaining access to services for PWUD who are homeless was a lifeline during the pandemic [69]. Another subsequent case study, conducted in Dublin (Ireland), where health services specifically expanded harm reduction services for people who were homeless during the pandemic, resulted in improved access to MAT and Naloxone (opioid overdose reversal drug) and the home delivery of key medications for PWUD (e.g., methadone and benzodiazepines). The authors concluded that the pandemic provided a basis on which to successfully remove regulatory obstacles and improved the political will to improve access to these lifesaving interventions [30]. It is important that future research examines the outcomes of all these measures and that in the event of another pandemic that the evidence base for these additional measures is known. 

Only one included study examined the risks of contracting COVID-19 amongst PWUD and found that people with a recent diagnosis of SUD (within the past year) were at increased risk of contracting COVID-19 compared to people without that diagnosis. This study also found that people with opioid use disorder (OUD) and those who were African American had increased odds of contracting COVID-19 and therefore are at higher risk for adverse outcomes [46]. While no other included studies examined the odds of contracting COVID-19 amongst people with SUD, there is a growing body of research that has shown that PWUD may be at greater risk of acquiring COVID-19, due to less ability to socially distance [70] and the increased risk of ‘non-White’ groups of contracting COVID-19 [71,72]. 

PWUD who are incarcerated are another important vulnerable group but only one study conducted in a prison setting met our inclusion criteria [49] and found reductions in the provision of OAT. Reducing access to ‘life saving’ medications such as OAT may impact negatively on an already vulnerable population. There is already good evidence of the effectiveness of OAT in prisons in preventing overdose, drug-related deaths [73] and in reducing mortality and improving other post imprisonment outcomes [73,74] and so maintenance of this access during a pandemic is particularly important.

### 4.3. Reduced Access to Treatment and Harm REDUCTION Services

Several studies reported reduced access to harm reduction services owing to the pandemic [48,52,61] though only one study inferred that this was related to a subsequent rise in DRDs [41]. More recent research has provided further evidence of the impacts of reduced service access including reductions in uptake of injecting equipment and MAT as well as a reduction of uptake of HIV and other BBV testing in Spain, UK and Sweden [75,76,77]. However, one recent study conducted in Sweden [76], which unlike many other countries did not impose a general societal lockdown, reported that harm reduction services were accessed as usual (at worst) during the initial stages of the pandemic and that distribution of needles and syringes increased. DRDs were found to have decreased over the period of study. In addition, this study showed that prevalence of SARS-CoV-2 was low amongst PWUD compared to the general population. These are noteworthy findings and suggest that although PWUD may have heightened risk factors for acquiring COVID-19, specific governmental and service responses—such as keeping services open (as best as possible)—may mitigate these risk factors. This is an important consideration for other jurisdictions.

Other mitigation measures that were introduced during periods when social restrictions were in place were increasing dispensing of take-home medications such as methadone. In the results section we noted that two of the included studies examined increases in the provision of take-home medication (OAT) during the initial stages of the pandemic and found there to be few negative consequences associated with this in terms of diversion of medication [52,53,54]. These are interesting findings as the issue of non-supervised consumption of OAT is a contested topic and especially relating to concerns over the diversion of medication and potential increased risk of overdose [78]. Research on this issue conducted prior to the pandemic has found that safe storage of these medicines at home can be problematic and can also pose a risk to others living in the household (e.g., children) [79], and that safety guidance is sometimes neither adequately presented nor adequately recalled by PWUD potentially exacerbating problems with safe storage [79,80]. A recently published paper based on interview data with PWUD during the pandemic found that the diversion of medication was used as a means of helping other PWUD to avoid withdrawals as well as to provide other sources of finance when social restrictions had educed opportunities for financing for PWUD [78]. Further research is required on the efficacy of this approach, including outcomes, to enable planning for future service provision including further lockdowns and pandemics. 

### 4.4. Drug Related Deaths 

Arguably the ‘strongest’ studies included in our review were those that we grouped under ‘emergency medical settings’ [38,40,44,45,55]. All were conducted in the USA and most used very large secondary data sets which suggested that both fatal and non-fatal drug overdoses had increased in the early stages of the pandemic. Two studies that examined ambulance call data showed that overall calls (or ambulance runs) relating to drug overdoses increased after the pandemic began but also that there was an increase in instances of refusals of transport for substance-related calls [40,45]. These studies raise concerning issues that also require further research but if either of these key findings are replicated elsewhere then the consequences for PWUD and the ongoing public health emergency of drug-related deaths will continue to escalate. One large scale qualitative study of the experiences of PWUD in Canada suggests that the risk of fatal and non-fatal overdose may have been elevated in part because of PWUD being compelled to use drugs other than their usual drug(s) of choice because of the disruptions to routes of drug supply in the pandemic [81]. In essence, switching to different types of drug use can lead to a reduction in drug tolerance and put people at greater risk of overdose [81]. Further investigation of what might explain the causes of increases in drug overdoses, and how best to mitigate them, during the pandemic is also required as a matter of urgency. While the reasons for this were not greatly discussed in our included papers, one paper did associate the increase in DRDs with reduced access to harm reduction services [41]. Given the known effectiveness of MAT and naloxone in preventing DRD [3,82] then reductions and/or new barriers to accessing these life-saving interventions could indeed feasibly lead to increases in fatal drug overdose and it would seem apt to conclude that ensuring the continuation of these critical services in future would be an important mitigation measure during any future pandemic. 

### 4.5. Drug Use Patterns 

Only three of the studies included examined impacts on drug use per se and each found a different result: one reported a decrease in drug use, one reported no change or a decrease in drug use and one reported an increase, thus presenting an inconclusive picture on the true impact [50,51,59]. None of our papers included research on novel psychoactive substances but there is evidence some countries have seen increases in the use of these drugs over the course the pandemic [83,84]. Nevertheless, this inconclusive evidence relating to whether drug use has increased or decreased has subsequently been supported in a trans-European study (seven countries) using wastewater analysis to examine the impact of ‘stay at home orders’ measures on specific stimulant and cannabis use. This study found that the picture was heterogenous rather than uniform indicating that potential responses to changes in drug use patterns are therefore hard to predict [85]. 

### 4.6. Answering the Research Questions

This review set out with four specific review questions. The answers to these are the following:

#### 4.6.1. Question 1. What Evidence Exists Regarding the Impacts of Any of the Novel Coronavirus Outbreaks of the 21st Century on Problem Drug Users, including COVID-19, Drug-Related Deaths and Other Harms; What Is the Quality of the Evidence?

The only empirical evidence available in relation to our question about problem drug users relates to the COVID-19 pandemic and the quality of evidence is very limited, both in terms of the types of methods employed (mostly observational and some limited qualitative research) but also in terms of how well these methods were applied and reported. There is also limited empirical evidence on the impact of COVID-19 on drug harms and little data related specifically to drug deaths.

#### 4.6.2. Question 2. How Have Services Who Provide Support for PDU and Policy Makers Responded to Any of the 21st Century Outbreaks, including COVID-19 and What Is the Quality of the Evidence Supporting Their Responses?

There is limited empirical evidence relating to the responses of services and policy makers during the current pandemic and of the empirical data that do exist they mainly concern evaluations of service usage. However, although no empirical research on the impact of COVID-19 on services themselves met our inclusion criteria (i.e., was found at the time searches for this review were conducted), some of the papers included here as part of our discussion do refer to service reductions and some claim that reductions in service provision have impacted negatively on issues such as initiation of people on methadone for the first time and even have resulted in higher numbers of DRDs. 

#### 4.6.3. Question 3. What Are the Gaps in Evidence and What Are the Future Research Questions of Importance in Responding to Any Future Outbreaks?

Our review shows that there are many gaps in evidence regarding the impacts on PDU and service/policy responses. The gaps in research relate primarily to three key areas: to the impact on individuals, to the extent and impact of service changes; policy responses. Some of the key areas for research identified relate to the immediate and longer term impacts of COVID-19 (both directly and indirectly) on PWUD, including less researched or additionally vulnerable PWUD (e.g., women, BAME communities, people in recovery communities); what are the impacts of new modes of service delivery that have occurred since the pandemic and what are the unintended consequences of these; finally, what impact have policy decisions had on PWUD and on service staff and service provision? 

#### 4.6.4. Question 4. What Are the Implications of Past Responses to Coronavirus Epidemics/Pandemics to Inform Future Service and Policy Responses and Specifically in Scotland?

No empirical studies were found relating to PWUD concerning past responses in previous coronavirus epidemics/pandemics. 

## 5. Strengths and Limitations

The strengths of this review are that we included only empirical evidence and so therefore we excluded commentary and opinion pieces. In addition, we used a systematic and robust approach that details stages of the process and includes rigorous quality assessment. We also searched multiple sources to accommodate and account for the emergent nature of the pandemic and we published a prior protocol.

A limitation of the study relates in part to the limited range and quality of the included studies which in turn impact on providing detailed answers to our research questions. A second limitation is that we may have missed studies that were on-going, or unpublished at the time. This is particularly the case during the COVID-19 pandemic when many research funding bodies have specifically re-oriented (at least some) funding towards research on myriad aspects of the pandemic and hence increases the likelihood of there being on-going studies that we were not able to include.

## 6. Conclusions

The full impact of the current COVID-19 pandemic is not yet known, nor is its impact on people who use drugs and on the services which provide support and treatment to people who use drugs. However, our review of relevant empirical studies conducted up until the end of October 2020 highlights that few empirical studies have been conducted to examine the impacts on or experiences of PWUD. No studies were found that addressed these issues during the SARS-Cov-1 or MERS coronavirus epidemics.

The range of study types is narrow: most are observational and only few are qualitative. Only two qualitative studies were located. Overall, the quality of included studies was not high—most were judged as satisfactory or inadequate.

We conclude that the empirical evidence is generally not of sufficient quality to inform future responses to further COVID-19 outbreaks or indeed other novel coronavirus outbreaks but we have noted where consideration may be given to some interventions such as maintaining access to evidenced based interventions (e.g., MAT and naloxone) as well as a need for additional supports for more vulnerable PWUD such as people who are homeless, who have additional comorbidities and from BAME groups. There remains a large gap in knowledge that is necessary to inform future responses.

## Figures and Tables

**Table 1:** Inclusion and Exclusion criteria.

Inclusion	Exclusion
Studies reporting any impact on people who use drugs (PWUD) due to Severe Acute Respiratory Syndrome (SARS), Middle Eastern Respiratory Syndrome (MERS) or COVID-19	
Studies reporting any service response to PWUD due to SARS, MERS or COVID-19	
Qualitative, quantitative, mixed methods or review papers	Non empirical studies, i.e., commentaries, editorials, opinion pieces
Published in English	Published in language other than English
Published between 2000 and October 2020	Studies published prior to 2000

**Table 2:** Components of the EPPI-Centre weight of evidence score.

WoE ^1^ Components	Definition	Scoring Range
Methodological Quality	The trustworthiness of the results judged by the quality of the study within the accepted norms for undertaking a particular type of research design	1 = excellent 2 = good 3 = satisfactory 4 = inadequate
Methodological Relevance	The appropriateness of the study design for addressing particular research questions	1 = excellent 2 = good 3 = satisfactory 4 = inadequate
Topic Relevance	The appropriateness of focus of the study design for addressing their particular research questions	1 = excellent 2 = good 3 = satisfactory 4 = inadequate

^1^ Weight of evidence.

**Table 3:** Characteristics of included studies (ordered by weight of evidence and setting relevance).

Study ID	Study Participants	Country	Setting	Setting Relevance	Method Quality	Method Relevance	Topic Relevance	Weight of Evidence
Qualitative studies								
DeJong 2020 [36]	Fifteen People with Substance Use Disorder (SUD) (11 under treatment and 4 in recovery)	Netherlands	Treatment and recovery	B	2	1	1	2
Pandit 2020 [37]	4 male patients with SUD	India	Community; addiction treatment centre, outpatient service	C	3	3	2	3
Quantitative studies								
Glober 2020 [38]	Calls for service (CFS) (all/suspected accidental overdose (OD)/suspected OD with naloxone administration/fatal OD)	USA	Emergency Medical Services (EMS) Indianapolis	B	2	2	1	2
Welle-Strand 2020 [39]	226 PWUD over 18 years.	Norway	User organisations near the open drug scenes and at low-threshold services	B	3	1	1	2
Slavova 2020 [40]	People brought to emergency services because of experiencing opioid overdose	USA	Emergency services for people who have an opioid overdose	C	2	2	1	2
Mariotinni 2020 [41]	All deaths occurring in Jan–Aug 2020 were included in which buprenorphine, amphetamines or THC-COOH were detected	Finland	Toxicology reports	C	2	1	1	2
Martinotti 2020 [42]	153 addicted patients, both outpatients and residential inpatients	Italy	Disorder (DSM-5) currently in treatment as outpatients and/or in a residency program as inpatients recruited across Italy. Under lockdown	C	3	2	1	2
Hochstatter 2020 [43]	64 People Living with HIV (PLWH) and Substance Use Disorders (SUD) (48 males, 15 females)	USA	Substance use and HIV care—Opioid Relapse Prevention and HIV Management	C	3	2	1	2
Ochalek 2020 [44]	Those presenting to emergency department with nonfatal unintentional overdose	USA	Emergency department	C	3	3	1	2
Weiner 2020 [45]	Those making ambulance calls for a substance misuse reason	USA	Ambulance	C	1	1	2	2
Wang 2020 [46]	73,099,850 unique patients, including 7,510,380 patients with a diagnosis with SUD (substance use disorders)	USA	Population-level electronic health record (EHR) from 360 hospitals and 317,000 providers across 50 states in the US since 1999	C	2	3	2	2
Starks 2020 [47]	455 CiS male 18 years+ who use drugs: marijuana, cocaine, crack, crystal methamphetamine, GHB, and Ketamine	USA	Cis male LGBTQ+ community	C	2	2	3	2
Whitfield 2020 [48]	People attending the 105 (91%) of the 115 sites providing NSP services (community pharmacies and specialist service sites)	UK	NSP across Cheshire and Merseyside	A	3	3	1	3
Bandara 2020 [49]	Wardens, sheriffs, medical directors and other leadership from 19 prisons	USA	Prison	C	3	2	1	3
Hawke 2020 [50]	622 youths aged 14 to 28 recruited across 4 existing participant cohorts	Canada	Based at Centre for Addiction and Mental Health, Toronto	B	3	2	3	3
Peacock 2020 [51]	People aged 18 or older who have used ecstasy and other illicit stimulants	Australia	Adults who have used ecstasy and other illicit stimulants	B	3	3	3	3
Seyed 2020 [52]	Unclear—aimed at substance use treatment and harm reduction services	Global	Substance use treatment and harm reduction services	B	3	3	2	3
Figgatt 2020 [53]	104 persons receiving methadone from three clinics	USA	PWUD receiving from 3 methadone clinics	C	3	3	1	3
Trujols 2020 [54]	Take home medication schedule (weeks) pre and post COVID-19 for 102 patients	Spain	Methadone Clinic Barcelona	C	3	3	2	3
Rodda 2020 [55]	Opiate, opioid, heroin, and/or fentanyl related overdose admission to emergency room	USA	Emergency room	C	3	3	2	3
Little 2020 [56]	Patients receiving first-time prescriptions for Medications for Opioid Use Disorder (MOUD)	USA	Prescriptions for Medications for Opioid Use Disorder	C	3	2	2	3
Mellis 2021 [57]	1148 PWUD and family	USA	Members of the Addictions Policy Forum	C	3	4	2	3
Rozanova 2020 [58]	123 older people with HIV (males and females). 42% had co-occurring Substance Use Disorders. Plus some (unspecified numbers) telephone interviews with HIV and addiction service providers	Ukraine	Older people with HIV receiving treatment for HIV	C	3	2	3	3
Wainwright 2020 [59]	75K + 75K (before and after COVID-19) Individuals (urine test results) with or at risk of SUD	USA	Nationwide lab urine results	C	2	3	4	3
Nelson 2020 [60]	6 community drop in centres for PWUD	Nigeria	Community drop in centres for PWUD in Nigeria	C	4	3	1	4
Malczewski 2020 [61]	Those working in drug-related services including Drug enforcement service; Prevention institution; Harm reduction; Drug treatments	Poland	Drug treatment services in Poland	C	4	3	3	4
Program Evaluation								
Martin 2020 [62]	27 homeless people residing in a municipal shelter	Spain	Homeless people in Salamanca city under lockdown in a municipal shelter	C	3	3	2	3

**Table 4:** Weight of evidence scores.

Components	Excellent	Good	Satisfactory	Inadequate
Methodological Quality	0	8	17	2
Methodological Relevance	4	9	13	1
Topic Relevance	12	9	5	1
Overall WoE ^1^	0	11	14	2

^1^ Weight of evidence.

**Table 5:** Treatment services/treatment/recovery.

Study	Method (Rating)	Participants	Setting (Context Rating)	Main Findings (Weight of Evidence—WoE)
*Qualitative*				
DeJong 2020 [36]	In-depth interview 2 Good	15 People with Substance Use Disorder (SUD) Outpatient (8); Inpatient (3); Stable Recovery (4); 11 men and 4 women; mean age 37.2 years	SUD clinical inpatient and outpatient practice or non-clinical projects (B) Netherlands (South East)	COVID-19 has had a serious impact on thoughts, feelings, and behaviours. Thoughts were associated with a range of negative feelings and behaviours, such as stress, anger, avoidance, and isolation. Those still in treatment were fighting against the temptation to start using again; they felt emotionally isolated and sometimes patronised by healthcare workers. (WoE: 2 Good)
Pandit 2020 [37]	Case studies 3 Satisfactory	4 males with SUD	SUD outpatient practice (C) India (no specific location)	Challenges for patients with opioid use disorders in this period, including increased risk of criminal charges due to increased policing, fear of contracting COVID-19 and transmitting it to family members, and stress due to occupational disruption and financial difficulties. Future policies should consider ways to enhance capability to provide medication-assisted treatment more easily to patients who have constrained resources, transport, skills, or willingness to quit. (WoE: 3 Satisfactory)
*Quantitative*				
Martinotti 2020 [42]	Questionnaire survey 3 Satisfactory	153 people with diagnosis of SUD in treatment; mean age 39.8 years; 78% males	Outpatients and/or residency program inpatients (C) Italy (various regions)	The presence of a moderate psychopathological burden correlated to poor quality of life and low craving scores. More than half of the cohort reported reduced quality of life during COVID-19 lockdown, and the analysis showed a negative correlation between perceived quality of life and reported craving. (WoE: 2 Good)
Figgatt 2020 [53]	Questionnaire survey 3 Satisfactory	104 persons receiving methadone from three clinics; aged 18 years and over; 56% of the sample were male; 90% of the sample classed as ‘non-Hispanic White’	Methadone prescribing clinics (C) USA (North Carolina)	Before COVID-19, the clinic-level percent of participants receiving any amount of days’ supply of take-home doses at each clinic ranged from 56% to 82%, while it ranged from 78% to 100% since COVID-19. The clinic-level percent of participants receiving a take-homes days’ supply of a week or longer (i.e., ≥6 days) since COVID-19 ranged from 11% to 56%. Among 87 participants who received take-homes since COVID-19, only four reported selling their take-home doses. (WoE: 3 satisfactory)
Radfar 2020 [52]	Questionnaire survey 3 Satisfactory	177 addiction medicine professionals from 77 countries	SUD treatment and harm reduction services (C) Global (77 countries)	Respondents from over 88% of countries reported that core medical and psychiatric care for SUDs had continued; however, only 56% of countries reported having had any business continuity plan, and 37.5% of countries reported shortages of methadone or buprenorphine supplies. Participants of 41% of countries reported partial discontinuation of harm-reduction services such as needle and syringe programs and condom distribution. In total, 57% of overdose prevention interventions and 81% of outreach services also having been negatively impacted. (WoE: 3 Satisfactory)
Malczewski 2020 [61]	Questionnaire survey 4 Inadequate	Provincial drug experts from 71 institutions	Primarily treatment services and harm reduction programmes (C) Poland (Nationwide)	The coronavirus epidemic has considerably affected the Polish drug services, especially in terms of ensuring the continuity of services at the adequate level. The operation of inpatient clinics (residentials treatment) and drop-in centres (harm reduction daily centre) has been limited the most. It is worth noting that substitution treatment has seen a higher demand for its services, which was the consequence of opioid users being deprived of income as a result of the epidemic. The lack of financial resources made drug users decide to enter opioid substitution treatment. (WoE: 4 Inadequate)
Whitfield 2020 [48]	Service provision monitoring 3 Satisfactory	People attending the 105 (91%) of the 115 sites providing NSP services	Needle and Syringe Programme provision (A) England (North West)	COVID-19 related restrictions resulted in the number of NSP clients decreasing by 36%, visits by 36%, and needles distributed by 29%. NSP coverage for those injecting psychoactive drugs halved, declining from 14 needles per-week during the 4 weeks to 15 March 2020 to 7 needles per-week by mid-April, and coverage has remained at this level since then. (WoE: 3 Satisfactory)
Trujols 2020 [54]	Before and after service evaluation 3 Satisfactory	102 attendees of methadone clinic	Methadone Clinic (C) Spain (Barcelona)	Take home medication (number of days provided) overall increased significantly [t(101) = −7.759, *p* < 0.001, d = 0.7680] comparing preCOVID-19 (6–12 March) vs. postCOVID-19 (13 March–12 May). This increase did not lead to any detectable signs that patients might be misusing/diverting medication. (WoE: 3 Satisfactory)

**Table 6:** Emergency medical services.

Study	Method	Participants	Setting	Main Findings
Glober 2020 [38]	Analysis of data from emergency medical services (EMS) system and Coroner’s office pre and post stay at home order 2 Good	Calls to emergency medical services with suspected overdose or where naloxone was administered or death by overdose (actual number not reported); participants were predominately white (67 and 71% pre and post); 26 and 30% black (pre and post); the remainder described as ‘other’. The majority of participants were aged under 40 both pre and post.	Emergency medical services drug overdose and drug deaths (B) USA (Indiana)	Data regarding emergency medicine calls for service (CFS) and suspected accidental drug overdose deaths were analysed. Overdose CFS and EMS naloxone administration showed an increase with the social isolation of the Indiana stay-at-home order, but a continued increase after the stay-at-home order was terminated. Despite a mild 4% increase in all EMS CFS, overdose CFS increased 43% and CFS with naloxone administration increased 61% after the stay-at-home order. Deaths from drug overdoses increased by 47%. (WoE: 2 Good)
Ochalek 2020 [44]	Before and after comparison of electronic medical record data (early months of the pandemic compared to the previous year) 3 Satisfactory	329 records of nonfatal, unintentional opioid-related opioid overdoses; the mean ages were 42.2 years and 44.0 years, 71 (70%) and 165 (73%) were male, 64 (63%) and 181 (80%) were Black, respectively.	Urban emergency department (C) USA (Virginia)	Number of unintentional opioid-related opioid overdoses increased *(n* =102 March–June 2019; n = 227 March June 2020). In March through June 2019 and March through June 2020, 55 (54%) and 127 (56%) patients received a naloxone prescription and 45 (44%) and 154 (68%) received treatment resources and/or a referral at discharge, respectively. However, only 4 (4%) and 14 (6%) of the 17 (17%) and 46 (20%) admitted patients received an addiction medicine consult, and 3 (3%) and 23 (10%) accessed treatment at the outpatient clinic after overdosing, respectively. (WoE: 2 Good)
Rodda 2020 [55]	Electronic medical record data before and after social distancing mandates were enacted 3 Satisfactory	189 patients seen for opioid-related overdose.	2 urban emergency departments (C) USA (San Francisco)	Emergency departments saw approximately 2.5 patients per day with opioid overdose, compared with 1.4 patients per day prior to this period. From 16 March to 18 April, there were 1.47 deaths per day, compared with 0.95 deaths per day prior to this period. During the first weeks of a COVID-19 pandemic, emergency room presentations and deaths related to opioid overdose may increase during an isolation period. (WoE: 3 Satisfactory)
Slavova 2020 [40]	Analysis of ambulance runs resulting in visits to Emergency Room before and after onset of COVID-19 pandemic 2 Good	Emergency response records of patients involved in ambulance runs for opioid overdose; no patient demographic data.	State Ambulance Reporting System (C) USA (Kentucky)	EMS runs in response to opioid overdoses have significantly increased since the COVID-19 crisis began. By comparing the period before the emergency declaration was made to the period after the declaration, EMS runs for opioid overdose have increased both in the rate of transportation to ED and, critically, in the number of those who were treated on the scene and refused transportation to ED. (WoE: 2 Good)
Weiner 2020 [45]	Retrospective cross-sectional analysis of evaluating average daily 9-1-1 ambulance calls for substance use-related issues compared with all other calls 2 Good	All 9-1-1 ambulance calls from February 15, 2020 to 15 May 2020; mean age 51.6 years (pre COVID-19) and 52.6 years post COVID-19); males were 49.8% of the pre sample and males and 52.8% of the post sample.	9-1-1 ambulance calls (C) USA (Massachusetts)	Calls for substance-related reasons decreased by 16.4% compared with prior to the state-wide emergency. However, despite an initial decrease in calls, after the stay-at-home advisory calls for substance use began increasing by 0.7 (95% confidence interval (CI) 0.4–1.1) calls/day, while calls for other reasons did not significantly change (þ1.2 (95% CI −0.8 to 3.1) calls/day). Refusal of transport for substance-related calls increased from 5.0% before the state-wide emergency to 7.5% after the declaration (*p* < 0.001). (WoE: 2 Good)

**Table 7:** Low threshold services/shelter/homeless.

Study	Method	Participants	Setting	Main Findings
Martin 2020 [62]	Programme evaluation with interview data 3 Satisfactory	29 homeless people (a proportion of whom had substance misuse disorder or psychiatric disorder); 27 evaluated; 67% male; ‘average’ age 37 years; 33% had a SUD.	Hostel for confined homeless of the city council social services (C) Spain (Salamanca)	Due to the pandemic caused by COVID-19, social and healthcare circumstances relative to homeless population, which usually are complicated, could be even more difficult. However, due to the intervention and implementation of this new program in the City Hall resources made by the Psychiatry Service, the objectives of detecting, treating and referring patients to social and mental health care resources, turned the unfortunate situation of the pandemic into an opportunity for this population. (WoE: 3 Satisfactory)
Nelson 2020 [60]	Questionnaire survey collecting service level data 4 Inadequate	Managers of drop-in centres (n not reported).	Drop-in centres for PWUD operated by Non-Governmental Organizations (C) Nigeria (4 regions)	The lockdown limited the range and quality of services provided, and constrained uptake by PWUD. Service utilisation declined from 375 users in October 2019 to 198 in April, before reaching 321 in May. Female users were more affected by the disruption than men. Cannabis was the drug most commonly used by service users followed by opioids and alcohol. There were significant gaps in service provision, including limited face-to-face counselling and discontinuation of other services. (WoE: 4 Inadequate)
Welle-Strand 2020 [39]	Questionnaire survey 3 Satisfactory	226 PWUD attending COVID-19 isolation units; mean age 43.1, 73% males.	Isolation units for COVID-19 positive PWUD (B) Norway (3 cities)	The main finding was that current or recent OMT experience (i.e., treatment engagement) was associated with improved knowledge of common COVID-19 symptoms and about available services. OMT may play an important role in COVID-19 prevention, as current and previous OMT patients were more likely to be aware of COVID-19 symptoms, as well as COVID-19 services available for PWUD. (WoE: 2 Good)

**Table 8:** People who use drugs/with diagnosis of Substance Use Disorder in predominantly community settings.

Study	Method	Participants	Setting	Main Findings
Hawke 2020 [50]	Questionnaire survey 3 Satisfactory	622 participants aged 14–28; 62% female; 61% Caucasian	4 existing participant cohorts based at the Centre for Addiction and Mental Health (CAMH) (C) Canada (Toronto, Ontario)	Reports of pre-pandemic mental health compared to intra-pandemic mental health show a statistically significant deterioration of mental health across clinical and community samples (*p* < 0.001), with greater deterioration in the community sample. A total of 68.4% of youth in the clinical sample and 39.9% in the community sample met screening criteria for an internalising disorder. Substance use declined in both clinical and community samples (*p* < 0.001), although 23.2% of youth in the clinical sample and 3.0% in the community sample met screening criteria for a substance use disorder. Participants across samples report substantial mental health service disruptions (48.7% and 10.8%) and unmet support needs (44.1% and 16.2%). (WoE: 3 Satisfactory)
Mellis 2020 [55]	Questionnaire survey 3 Satisfactory	1,148 members of the APF network of patients, families and survivors; aged 18 years and over; 66% female, 88% Caucasian	Addiction Policy Forum (APF) fielded an uncompensated survey via email to their national (U.S.) network of patients, families, and survivors (C) USA (multiple states)	Individuals who reported a history of use of multiple substances were more likely to report that COVID-19 has affected their treatment and service access and were specifically more likely to report both use of telehealth services and difficulties accessing needed services. These findings suggest that individuals with a history of using multiple substances may be at greater risk for poor outcomes due to COVID-19, even in the face of expansion of telehealth service access. (WoE: 3 Satisfactory)
Peacock 2020 [51]	Questionnaire survey 3 Satisfactory	389 people aged 18 or older who have used ecstasy and other illicit stimulants at least once monthly in the preceding six months Median age 23; 65% males	Participants recruited from the community via social media and/or peer referral (B) Australia (multiple states)	(Preliminary results). Most participants reported no change or a decrease in their drug use since COVID-19 restrictions compared to before March 2020, although changes in use varied by drug. Perceptions of drug availability were mostly that it remained stable. Participants reported negative impacts on mental health but did not report difficulties engaging with services for alcohol and drug-related reasons and had sought information about practices to reduce the risk of COVID-19 transmission while using drugs. (WoE: 3 Satisfactory)
Wainwright 2020 [59]	Cross sectional study of urine drug tests before and during COVID-19 declaration 2 Good	150,000 patient specimens of urine sent for testing; aged 18 years and over; 47% male. No ethnicity information declared	Healthcare settings where practitioners have patients who are diagnosed with or at risk of substance misuse (C) USA (multiple states)	This study demonstrated that urine drug test positivity in a population diagnosed with or at risk of substance use disorders increased significantly for illicit cocaine, fentanyl, heroin, and methamphetamine from the 4 months before the COVID-19 emergency declaration to the 4 months after the COVID-19 declaration. (WoE: 3 Satisfactory)
Wang 2020 [46]	Retrospective case–control study of electronic health records 2 Good	73,099,850 unique patients, of whom 12,030 had a diagnosis of COVID-19. 722,370 had been recently diagnosed with SUD; includes all age ranges; predominantly Caucasians	Electronic health records from 360 hospitals and 317,000 providers across 50 states in the US since 1999 (C) USA (50 states	Patients with a recent diagnosis of SUD (within past year) were at significantly increased risk for COVID-19 (adjusted odds ratio or AOR = 8.699 [8.411–8.997], P < 10−30). Compared to patients without SUD, patients with SUD had significantly higher prevalence of chronic kidney, liver, lung diseases, cardiovascular diseases, type 2 diabetes, obesity and cancer. Findings identify individuals with SUD, especially individuals with OUD and African Americans, as having increased risk for COVID-19 and its adverse outcomes, highlighting the need to screen and treat individuals with SUD as part of the strategy to control the pandemic while ensuring no disparities in access to healthcare support. (WoE: 2 Good)
Little 2020 [54]	Predictive linear regression of Electronic Health Care population data 3 Satisfactory	Data are pooled from 16 healthcare organisations that span 11 states and cover 11.1 million patients	Receipt of prescription of medications for opioid use disorder (C) USA (12 states)	The number of patients receiving first-time prescriptions for Medications for Opioid Use Disorder (MOUD) decreased by over 30% in the spring of 2020 when compared to trends observed in EHR data from January 2017 to May 2020. This finding suggests that patients at risk for opioid use disorder (OUD) and overdose are increasingly vulnerable during the COVID-19 pandemic. (WoE: 3 Satisfactory)
Mariotinni 2020 [41]	Before and after lockdown analysis of post-mortem toxicology cases 2 Good	All post-mortem toxicology cases in Finland	Post-mortem where buprenorphine, amphetamine or cannabis was found (C) Finland	Immediately after government restrictions in March 2020, the numbers of buprenorphine, amphetamine and cannabis findings increased. The increase was most noticeable for amphetamine and was evident in all age groups. Findings indicate by association that there is an increased risk of drug-related harm (including death) in Finland. (WoE: 2 Good)

**Table 9:** People with HIV and Substance Use Disorder.

Study	Method	Participants	Setting	Main Findings
Hochstatter 2020 [43]	Questionnaire survey 6 weeks before national emergency compared to 6 weeks after 3 Satisfactory	60 individuals with SUD and HIV; 75% male; 59% black or African American, 34% White, 2% mixed; 5% ‘other’	Opioid relapse prevention and HIV management mobile-health Intervention (C) USA (Wisconsin)	During the pandemic, people who live with HIV and SUD increased illicit substance use and contact with other substance-using individuals and decreased their confidence to stay sober and attend recovery meetings. The proportion of people missing their HIV medications also increased, and confidence to attend HIV follow-up appointments decreased. (WoE: 2 Good)
Rozanova 2020 [58]	Questionnaire survey May 2020 3 Satisfactory	123 older people (aged >50) with HIV and SUD; 47% women, also HIV and addiction treatment providers at frontlines and senior executive levels	Services providing treatment and care for older people with HIV and SUD (C) Ukraine (Kyiv)	While older people with SUD maintained HIV and SUD therapy throughout COVID-19 lockdown, social support is critical to avoiding treatment interruption. COVID-19 lockdown may disrupt MAT and ART among older people with HIV and SUD not only while being in place, but also during the reopening. After recent increases of support by clinicians, subsequent reduction of support may lead to people feeling even more isolated. (WoE: 3 Satisfactory)

**Table 10:** Sexual minority men.

Study	Method	Participants	Setting	Main Findings
Starks 2020 [47]	Matched cohort-controlled study with questionnaire survey compared respondents (surveyed May 6–17, 2020) and a matched sample selected from 65,707 respondents surveyed pre-COVID-19 2 Good	455 adult sexual minority CIS males who use drugs; age range 18+; 47% ‘white’; 29% ‘black’; 14% Latino; 10% other; drugs mainly marijuana	Geosocial networking apps for gay, bi, trans, and queer people seeking sexual partners. The COVID-19 cohort responded to an advertisement that included an image of one or more adolescent or adult males. (C) USA (multiple states)	While the proportion of participants reporting marijuana and other illegal drug use as well as CAS with casual partners declined during COVID, the association between other illegal drug use and sexual risk behaviour was amplified. (WoE: 2 Good)

**Table 11:** Prison setting.

Study	Method	Participants	Setting	Main Findings
Bandara 2020 [49]	Questionnaire survey 3 Satisfactory	Wardens, sheriffs, medical directors, and other leadership (no demographic data)	19 prisons (14 county jail systems and 5 state-level systems) that provide methadone and/or buprenorphine treatment for incarcerated populations (C) USA (multiple states)	Ten out of 16 systems reported downsizing their OAT programs. Seven of 16 systems made changes to medication dispensation processes. Half of systems report challenges implementing physical distancing (n¼8), and/or obtaining personal protective equipment (n¼8). In 13 out of 16 systems some OAT program participants were released early due to COVID-19 infection risk. (WoE: 3 Satisfactory)

## Data Availability

Data is contained within the article or Supplementary Material.

## Supplementary Materials

The following are available online at https://www.mdpi.com/article/10.3390/ijerph18168470/s1, File S1: PRISMA Checklist; File S2: Full Search Architecture; File S3: Results of Assessment of Methodological Quality.
