# Supplementary material for: Understanding the Impacts of Novel Coronavirus Outbreaks on People Who Use Drugs: A Systematic Review to Inform Practice and Drug Policy Responses to COVID-19
Source: Int J Environ Res Public Health. 2021 Aug 11;18(16):8470. doi: 10.3390/ijerph18168470 (PMC8394531; doi:10.3390/ijerph18168470)
Supplement: Supplementary file 1 [file ijerph-18-08470-s001.zip › Supplementary File S2 Search Architecture-final .pdf]

Supplementary File 2: Full search architecture

1. COVID-19 OR CORONAVIRUS OR 2019-ncov
2. SARS-CoV-2
3. SARS-CoV-1
4. MERS
5. SARS
6. 1 OR 2 OR 3 OR 4 OR 5
7. Problem Drug Users OR People who use drugs OR people who inject drugs
8. 6 AND 7
9. Limit 8 to English language; limit 8 to year range 2000 to 2020

**DATABASE(S)**	**Hits**	**Location**
Medline, Embase, PsycINFO	1,661	Endnote file
ASSIA	28	Word document
CinAHL	143	Endnote file
**Total**		

Medline/Embase/PsycINFO

1. COVID-SARS -MERS

exp Coronavirus Infections/ OR exp Coronavirinae/ OR exp Middle East Respiratory Syndrome Coronavirus/ OR exp SARS Virus/ OR COVID-19 OR severe acute respiratory syndrome OR novel coronavirus disease OR COVID-19 pandemic OR COVID-19 virus infection OR coronavirus disease-19 OR novel coronavirus infection OR 2019-nCoV infection OR coronavirus disease OR nCoV disease OR COVID-19 virus disease OR nCoV OR Wuhan coronavirus OR SARS-CoV-2 OR novel coronavirus OR COVID-19 virus OR coronavirus disease 2019 virus OR COVID19 virus OR Wuhan seafood market pneumonia virus OR SARS-CoV-1 OR Middle East respiratory syndrome

2. People who use drugs

exp Substance-Related Disorders/ or exp drug dependence/ or exp Drug Misuse/ or exp Prescription Drug Misuse/ OR exp Illicit Drugs/ OR Needle-Exchange Programs/ or Substance Abuse, Intravenous/ or Needle Sharing/ or (drug$ or polydrug$ or psychotropic$ or substance$) adj3 (abstain$ or abstinen$ or abus$ or addict$ or excessive or criminal or depend$ or habit$ or illegal$ or illicit$ or intoxicat$ or misus$ or nonprescri$ or non prescri$ or over dos$ or overdos$ or recreation$ or unlawful$ or withdraw$)

ASSIA

(People who use drugs OR MAINSUBJECT.EXACT.EXPLODE("Drug replacement therapy") OR MAINSUBJECT.EXACT.EXPLODE("Drug culture") OR MAINSUBJECT.EXACT.EXPLODE("Drug

dependency") OR MAINSUBJECT.EXACT.EXPLODE("Drug abuse") OR MAINSUBJECT.EXACT.EXPLODE("Drug abusers") OR MAINSUBJECT.EXACT.EXPLODE("Drug addiction") OR MAINSUBJECT.EXACT.EXPLODE("Drug related problems") OR MAINSUBJECT.EXACT.EXPLODE("Drug addicts") OR MAINSUBJECT.EXACT.EXPLODE("Drug dependency units")) AND (Covid OR novel coronavirus disease OR COVID-19 pandemic OR COVID-19 virus infection OR coronavirus disease-19 OR novel coronavirus infection OR 2019-nCoV infection OR coronavirus disease OR nCoV disease OR COVID-19 virus disease OR nCoV OR Wuhan coronavirus OR SARS-CoV-2 OR novel coronavirus OR COVID-19 virus OR coronavirus disease 2019 virus OR COVID19 virus OR Wuhan seafood market pneumonia virus OR SARS-CoV-1 OR Middle East respiratory syndrome) Limits applied

CinAHL

S10	S4 AND S9
S9	S5 OR S6 OR S7 OR S8
S8	TX COVID-19 OR severe acute respiratory syndrome OR novel coronavirus disease OR COVID-19 pandemic OR COVID-19 virus infection OR coronavirus disease-19 OR novel coronavirus infection OR 2019-nCoV infection OR coronavirus disease OR nCoV disease OR COVID-19 virus disease OR nCoV OR Wuhan coronavirus OR SARS-CoV-2 OR novel coronavirus OR COVID-19 virus OR coronavirus disease 2019 virus OR COVID19 virus OR Wuhan seafood market pneumonia virus OR SARS-CoV-1 OR Middle East respiratory syndrome
S7	(MH "SARS Virus") OR (MH "Severe Acute Respiratory Syndrome")
S6	(MH "Middle East Respiratory Syndrome Coronavirus") OR (MH "Middle East Respiratory Syndrome")

S5	(MH "Coronavirus+") OR (MH "Coronavirus Infections+") OR (MH "COVID-19") OR (MH "Middle East Respiratory Syndrome Coronavirus")
S4	S1 OR S2 OR S3
S3	(drug* or polydrug* or psychotropic* or substance*) n3 (abstain* or abstinen* or abus* or addict* or excessive or criminal or depend* or habit* or illegal* or illicit* or intoxicat* or misus* or nonprescri* or non prescri* or over dos* or overdos* or recreation* or unlawful* or withdraw*)"
S2	(MH "Substance Abuse, Intravenous") OR (MH "Substance Abuse+")
S1	(MH "Street Drugs") OR (MH "Drugs, Non-Prescription") OR (MH "Intravenous Drug Users") OR (MH "Drug Rehabilitation Programs")
